# Acute poisoning in children in Ethiopia: a cross-sectional study

**DOI:** 10.1038/s41598-022-23193-x

**Published:** 2022-11-05

**Authors:** Yalew Melkamu Molla, Kassahun Denekew Belachew, Gashaw Walle Ayehu, Assefa Agegnehu Teshome

**Affiliations:** 1 Department of Pediatrics and Child Health, College of Medicine and Health Science, University of Gondar, Gondar, Ethiopia; 2 Department of Biomedical Science, College of Health Science, Debre Tabor University, Debre Tabor, Ethiopia

**Keywords:** Diseases, Health care, Medical research

## Abstract

Acute poisoning is a global pediatric emergency problem. However, a wide variation in patterns of acute poisoning and associated factors across different geographical regions was stated. As a result, our research focused on the investigation of acute poisoning in children. An Institutional-based Retrospective study design was conducted at the University of Gondar comprehensive specialized hospital from October 2016 to October 2020. The analysis of the data was performed via SPSS Version 25. Furthermore, multiple logistic regression analysis was recruited. A P-value ≤ 0.05 was declared as statistically significant. Our study's prevalence of acute poisoning was 82/5489 (1.5%). 53 (64.6%) of patients were males. Of the total patients who had the diagnosis of poisoning, 54 (65.9%) came from rural areas. Venom was the most frequent offending agent (26.8%) and oral ingestion was the most common route of exposure (70.7%). Of the total patients who had the diagnosis of poisoning, 54 (65.9%) came from rural areas. Venom was the most frequent offending agent (26.8%), followed by insecticides (organophosphates) (21.5%). Accidental poisoning was the most common mode of poisoning more often than intentional (75.6–24.4%). The digestive tract (oral ingestion) (69.5%) was the commonest route of poisoning, followed by the cutaneous (skin bite) (24.4%). Death was three times more likely in the rural population than in urban residents [AOR 2.9 (1.21–13.7); P value 0.046]. Appropriate emergency care is the mainstay of the supportive management protocol for childhood poisoning.

## Introduction

Poisoning can be defined as the exposure of an individual to a substance that can cause symptoms and signs of organ dysfunction that eventually results in injury or death^1^. Acute poisoning means exposure to poison for a short period (less than 24 h) via any route. It can be intentionally or unintentional. It is the common cause of emergency admission, which may result in morbidity and mortality^2^. One systematic review and meta-analysis conducted in Ethiopia revealed that the mortality rate of acute poisoning was in the range of 0–14.8%^3^.

A serious global public health issue is acute poisoning, especially in youngsters^4^. Acute poisoning remains a prevalent medical emergency in the pediatric population despite extensive teaching programs and public awareness initiatives to prevent it^5^. Even amongst various geographical locations within the same country, the pattern of incidence and risk factors for acute poisoning is changing over time and differs from country to country^6^.

Insecticides, industrial technology, and medical pharmacology have all made significant advancements in the last few decades in the domains of agriculture^7^. The patterns of acute poisoning have seen noteworthy modifications in tandem with these advancements. Therefore, about 2% of all injury-related child deaths in poor nations were attributed to accidental poisoning^8^.

Information on the types, characteristics, and severity of poisoning in a particular area is crucial for both diagnosis and treatment as well as for the introduction of preventative measures in the future^9^. The epidemiological data and general information about the prevalence of poisoning and its particular short-term outcomes are unknown in Ethiopia. The few investigations conducted in other parts of the nation have not evaluated the pattern and immediate consequences of the reported poisonings^10^.

This study was carried out to assess the patterns, associated factors, and short-term outcomes of poisoning among poisoned children’s presented to the University of Gondar comprehensive specialized hospital, Ethiopia.

## Result

### Sociodemographic characteristics of the study participants

**Figure 1:**
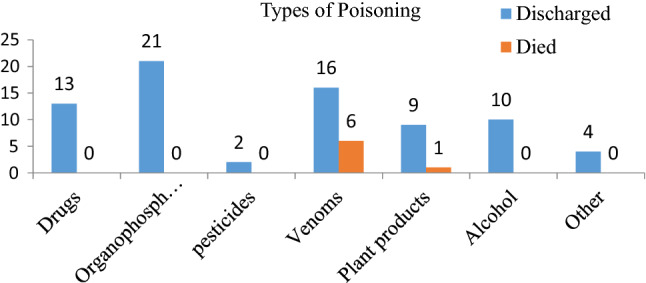
Overall distribution of poisoning cases with short-term outcomes.

The total pediatric emergency admitted patients were 5489, thus the prevalence of acute poisoning was 82/5489 (1.5%). In this study, the age range of all poisoned patients was from birth to 18 years of age. The highest proportion (42.7%) of the poisoning cases were in the age category of under 5 years of age. About 58 (70.7%) of the poisoned cases were males. In terms of where they lived, 65.9% of them said they lived in rural areas with their families. Regarding parental educational status, about 46 (56.1%) of them did not attend formal education (Table 1). Among the parents who did not attend formal education, 39 of them came from rural areas (Fig. 1).

**Table 1:** Sociodemographic characteristics of children with poisoning who were presented to the University of Gondar comprehensive specialized hospital from October 1, 2014 to October 1, 2020, North Gondar, Ethiopia.

Variable	Frequency	Percent
**Age in years**		
0–5	35	42.7
6–12	20	24.4
13–18	27	32.9
Total	82	100
**Sex**		
Male	53	64.6
Female	29	35.4
Total	82	100
**Residency**		
Urban	28	34.1
Rural	54	65.9
Total	82	100
**Educational status**		
Did not attend formal education	49	59.8
Elementary school	33	40.2
Total	82	100

### Type of poisoning agent, rout of exposure and manner of poisoning

**Figure 2:**
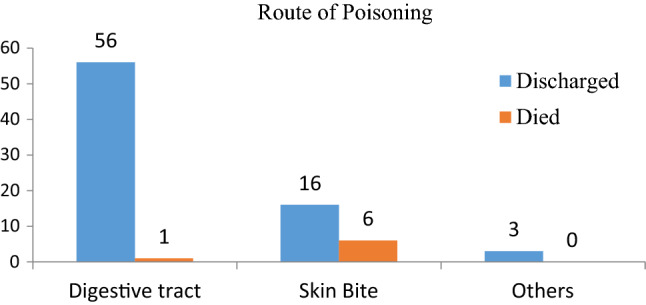
Overall distributions of poisoning cases by rout of ingestion with short-term outcomes.

**Figure 3:**
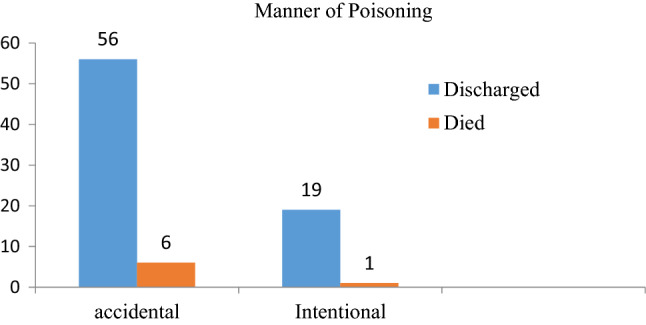
Overall poisoned case distributions by method of poisoning and their short-term outcomes.

All patients had a record of known poisoning agents, of which venom was the most common poisoning agent, 22/82 (26.8%) (Fig. 1). Of the total poisoned cases, oral ingestion was the most common route of exposure (58/82, or 70.7%), followed by cutaneous (skin bite) (22, or 26.8%) (Fig. 2). Moreover, accidental poisoning was the most common 62/82 (75.6%) manner of poisoning. Out of 20 intentional poisonings, 16 of them were given herbal medications for traditional healing purposes prior to hospital arrival (Fig. 3).

Most of the patients arrived at the hospital after one hour of exposure. Nausea, vomiting, diarrhea, and abdominal pain were the most common presenting symptoms at presentation, accounting for 82.9% in combination. Changes in mentation, bleeding, and respiratory symptoms were also responsible for 61%, 29.3%, and 18.3%, respectively (Table 2).

**Table 2:** Clinical characteristics of patients at the time of presentation at the University of Gondar comprehensive specialized hospital in Northwest Ethiopia (n = 82).

Clinical features	Frequency	Percent
**Time of arrival at the hospital**		
Within 1 h	8	9.8
Greater than 1 h	74	90.2
Nausea, vomiting, diarrhea, abdominal pain	68	82.9
Change in mentation	50	61
Headache, fatigue, weakness	6	7.3
Respiratory symptoms	15	18.3
Bleeding	24	29.3
Asymptomatic	2	2.4
Others(seizure)	12	14.6
**Past history of poisoning**		
Yes	2	2.4
No	80	97.6
**Quarrel before poisoning**		
Yes	16	19.5
No	66	80.5
**Unsafe storage of household poisons**		
Yes	9	11
No	73	89

### Duration of the hospital stay after arrival and outcomes at discharge

**Figure 4:**
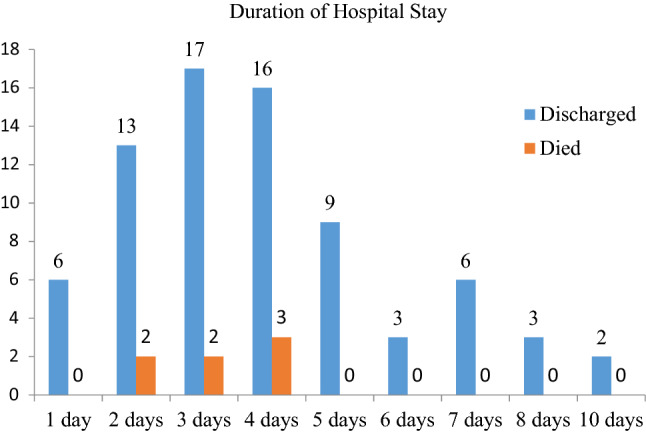
Overall distributions of poisoned children and their short-term outcomes over the course of their hospital stay.

The length of hospital stay after arrival was three or four days for most of the patients. The maximum hospital stay of the patients was 10 days. The current study showed that individuals who stayed more days at the hospital improved (Fig. 4).

Poisoning attempts were lower in the urban population than in the rural (34.1% in the urban and 65.9% in the rural). However, death was three times more likely in the rural population as compared to the victims who were urban residents [AOR 2.9 (1.2–13.8); P value 0.046] (Table 3).

**Table 3:** Factors associated with short-term outcome of acute poisoning (bivariate and multivariate logistic regression) among study participants at the University of Gondar Comprehensive specialized hospital, Northwest Ethiopia (n = 82).

Baseline information	Category	Short term outcome		AOR	95% CI	P-value
		Improved	Died			
Residency	Urban	26 (34.7)	2 (28.6%)	1	1	0.046
	Rural	49 (65.3%)	5 (71.4%)	2.9	1.2–13.8	
Sex	Males	48 (64%)	5 (71.4%)	1	1	0.094
	Females	27 (36%)	2 (28.6%)	0.3	0.1–0.9	
Age	0–1 year	6 (100%)	0 (0%)	1	1	0.746
	1–5 years	26 (89.7%)	3 (10.3%)	2.13	0.7–8.5	
	5–10 years	19 (95%)	1 (5%)	4.6	0.3–16.2	
	10–18 years	24 (88.9%)	3 (11.1%)	2.5	0.7–9.6	
Manner of poisoning	Accidental	56 (74.7%)	6 (85.7%)	1.8	0.9–3.4	0.20
	Intentional	19 (25.3%)	1 (14.3%)	1	1	
Way (route) of poisoning	Digestive tract	56 (74.7%)	1 (14.3%)	2.5	1.2–8.6	0.001
	Skin (BITE)	16 (21.3%)	6 (85.7%)	1.9	0.6–4.3	
	Others	3 (3.7%)	0 (0%)	1	1	
Time of arrival since exposure status	Within 1 h	8 (10.7%)	0 (0%)	1	1	0.004
	> 1 h	67 (89.3%)	7 (100%)	5.8	1.4–42.4	
Mental status at admission	Conscious	47 (62.7%)	3 (42.9%)	1	1	0.06
	Unconscious	28 (37.3%)	4 (57.1%)	1.0	0.3–3.4	
Antidote given	Yes	26 (34.7%)	6 (85.7%)	2.6	0.8–8.3	0.008
	No	49 (65.3%)	1 (14.3)	1	1	
Antibiotics given	Yes	7 (9.3%)	4 (57.1%)	1.41	1.6–5.3	0.002
	No	68 (90.7%)	3 (42.9%)	1	1	
Duration of hospital stay	≤ 48	36 (43.9)	4 (4.9)	3.5	1.1–11.5	0.002
	> 48	39 (47.6)	3 (3.7)	1	1	

*AOR* adjusted odds ratio, *CI* confidence interval.

## Discussion

Poisonings are a widespread public health issue that endangers the wellbeing of the general population and are one of the most frequent reasons for hospital admissions. Their yearly prevalence varies by nation, and even by province inside a single nation^11^. The current hospital-based retrospective study was conducted on the most difficult medical condition for which Ethiopia lacks adequate epidemiological clarification. It entailed looking into medical records of poisoning cases involving children (0–18 years) over 4 years.

According to Mowry et al., children under the age of six make up roughly half of all toxin exposure cases in the United States^12^. 60.68% of poisoning cases in India include children under the age of six, according to Bhat et al.^13^. According to Al-Barraq and Farahat, children under the age of five account for the bulk of child poisoning incidents in Saudi Arabia^14^. According to Oliveira and Suchara, children under the age of four in Brazil account for the majority of poisoning cases^15^. The results of all these studies were consistent with the findings of this research. The majority of the poisoning victims were aged between 0 and 5 years (42.68%), followed by ages between 11 and 18 years (accounting for 32.9%). This indicates that poisoning was most prevalent in children who were exposed to toxic agents accidentally and in adults who were largely intentionally exposed.

According to the current study, males are more likely than females to become poisoned as children. This is supported by several studies that have been conducted around the world^12,16,17^. The fact that males are more active than females might be one of the explanations for this. The precise cause, however, is unknown^16^.

In our study, most poisoned patients have low self or parental educational status. This is because most poisoned children are under 5 years old and most parents are from rural areas where educational access is actually low.

Our study showed that the number of poisoned children was higher in rural areas than that of urban areas (65.9% vs 34.1%), in which children poisoning admission rates have been consistently higher in rural areas than urban areas. This is probably due to the fact that parental educational status is low in rural areas (39 vs 7) and some poisons are more common in rural areas like insecticides.

Both accidental and intentional poisoning are possible. According to this study's findings, accidental poisoning accounts for 75.61% of all poisoning cases, which is consistent with research conducted by three pediatric referral hospitals in Addis Abeba^3^. The oral route was documented in the majority of poisoned cases (69.5%), which was similar to the results obtained at Jimma and Tikur Anbesa, Ethiopia^10,18^.

Venoms (snakebite) were the most common agent implicated in our poisoned patients. This could be explained by the fact that we are living in an area where snakes are abundant and poor practices of bite prevention and risk reduction like working in forests.

Organophosphate poisoning was the second most prevalent agent. These results were similar to those of other studies held in Jimma^10^. This could be explained by the fact that we are living in one of the major agricultural areas in Ethiopia, and the easy access and inappropriate usage of insecticides are common.

Rural residency was the independent factor of death treatment outcomes for acute poisoning, and it was approximately three times more likely to contribute to a death treatment outcome than urban residency. Furthermore, poisoning via the digestive tract is approximately 2.5 times more likely to result in a dying treatment outcome than other poisoning routes, since exposure status > 1 h increases the likelihood of a dying treatment outcome approximately 6 times more than 1 h of hospital arrival. Longer hospital stays may result in patients with more serious conditions, which may affect the outcome of their medical care—death.

The main drawback of this study is that it was retrospective, which led to missing patient data. Another limitation was that it was a single-center; retrospective study with a limited sample size, so findings might not be generalized.

Poisoning can be reduced through the use of effective prevention strategies by avoiding poisoning agents from the environment (e.g., removal of poisonous plants and removal of insecticides out of reach of children), enforcement of child-resistant packaging of necessary poisonous agents (e.g., medicines, household chemicals, and other toxins), and wearing protective equipment like shoes for snake bite. Hence, studies with larger samples and population-level data from both urban and rural areas could provide better estimates of the prevalence and risk factors for poisoning. Furthermore, it is essential to educate the public about poisoning prevention, early referral, and care. Despite the limitations mentioned above, we conducted a 6-year retrospective study to determine the prevalence and short-term outcome of poisoning in children. This study alarms researchers for further study at a multicenter level.

## Conclusion

It was noticed that compared to other reported statistics in Ethiopia, the rate of acute poisoning was highest in this study. The stakeholders should be encouraged by this issue to give the area more consideration when developing poisoning prevention and control strategies. It is necessary to manage and handle agrochemicals properly to reduce their harmful effects. Age, gender, and hospital stay length were all independent predictors of death treatment outcome. The authors advise conducting a prospective study in a multicenter area to identify the variables influencing bad treatment outcomes while taking into account clinical, laboratory, and therapy-related variables.

## Methods

This institutional based retrospective study on hospital records was conducted at the University of Gondar comprehensive specialized hospital from October 1, 2016 to October 1, 2020 over a 5-year period. The hospital is the teaching hospital and one of the famous Ethiopian institutes, giving different health services for the total population of North Gondar, which is 2,929,628 according to the census conducted in 2007. It is located in the northwest part of Ethiopia, which is 727 km away from Addis Ababa. It provides many services for the community, such as diagnosis and treatment of acute poisoning for those patients presented directly by themselves or referred from other hospitals. The patients had the service for their own payment.

All patients having a diagnosis of acute poisoning and who had presented at the University of Gondar Comprehensive specialized hospital with inclusion criteria were our study participants. The inclusion criteria were all acutely poisoned patients less than 18 years of age and who were present at this hospital from October 1, 2016–October 1, 2020 obtained by searching in the medical records manually. Patients with incomplete information on their medical charts would be excluded from the study. A total of 103 patients were seen due to acute poisoning, but because of incomplete information, 21 patients were excluded. Finally, 82 patients were included in this study for analysis.

The data collection instrument (checklist) was designed from the previous published articles with some modifications^2–4,9,10,18^. Then, a pre-test was done to check the consistency and completeness of the checklist depending on the objectives of the study. Three trained intern medical students did the actual data collection. A pretested structured checklist was used to collect all the accessible information from the patient's chart. From the patient's registration logs, the identification number of acute poisoning cases was noted. The data collectors then filtered the patient's chart from the medical record room using this identification number. Age, sex, residence, educational level, diagnosis, route, manner of poisoning, time of hospital admission since exposure, type of poisoning agent, given medication, consciousness at admission (conscious and unconscious), time of arrival, short-term outcome, and hospital stay were assessed.

Data quality assurance was performed prior to data entry and analysis to ensure that the data was complete, accurate, clear, and consistent. The data were entered into the epi Info version 7 program and then exported to the SPSS version 22 program for analysis. Sociodemographic and clinical-related data were used to describe the pattern and short-term outcomes of poisoning cases. As a measure of association, the adjusted odds ratio and 95% confidence interval were used to look for any associations between independent variables and outcome variables. Finally, text, tables, and graphs were used to present the study's findings. The significance level was established at a p-value of less than 0.05.

### Ethical approval and informed consent

The ethical review committee of the college of medicine and health sciences of the University of Gondar approved the study. This study used data from patients’ medical records. Patient data confidentiality was guaranteed. Therefore, informed consent from the study participants for their data was not required.

## Data Availability

The datasets generated during and/or analysed during the current study are available from the corresponding author on reasonable request.
